# Body mass index (BMI) may be a prognostic factor for gastric cancer with peritoneal dissemination

**DOI:** 10.1186/s12957-016-1076-1

**Published:** 2017-02-23

**Authors:** Shi Chen, Run-Cong Nie, Li-Ying OuYang, Yuan-Fang Li, Jun Xiang, Zhi-Wei Zhou, YingBo Chen, JunSheng Peng

**Affiliations:** 1 The 6th Affiliated Hospital, Sun Yat-Sen University, No. 26, YuanCun ErHeng Road, TianHe District 510655 Guangzhou, China; 2 Department of Gastropancreatic Surgery, Sun Yat-Sen University Cancer Center, 651 Dongfeng East Road, 510060 Guangzhou, China; 3 Department of Intensive Care Unit, Sun Yat-Sen University Cancer Center, 651 Dongfeng East Road, 510060 Guangzhou, China

**Keywords:** BMI, Prognosis, Gastric cancer, Peritoneal dissemination, Palliative chemotherapy

## Abstract

**Background:**

The aim of this study is to investigate whether body mass index (BMI) is a prognostic factor in gastric cancer patients with peritoneal dissemination.

**Methods:**

This is a retrospective study consisting of 518 patients with a histological diagnosis of gastric cancer with peritoneal dissemination seen at the Sixth Affiliated Hospital of Sun Yat-Sen University and Sun Yat-sen University Cancer Center between January 2010 and April 2014. Patients were followed until December 2015. Chi-square test and Kaplan-Meier survival analysis were used to compare the clinicopathological variables and prognosis.

**Results:**

Univariate analyses showed that significant prognostic factors included palliative gastrectomy (*p* < 0.001), tumor size (*p* < 0.001), tumor location (*p* = 0.011), peritoneal seeding grade (*p* < 0.001), ascites (*p* = 0.001), serum CEA level (*p* = 0.002), serum CA19-9 level (*p* = 0.033), palliative chemotherapy (*p* < 0.001), and BMI group (*p* < 0.001). For patients with palliative chemotherapy, univariate analysis revealed that palliative gastrectomy (*p* < 0.001), tumor size (*p* = 0.002), tumor location (*p* = 0.024), peritoneal seeding grade (*p* = 0.008), serum CEA level (*p* = 0.041), and BMI group (*p* < 0.001). Multivariate analysis revealed that BMI was an independent prognostic factor in gastric cancer patients with peritoneal dissemination, especially in patients who received palliative chemotherapy.

**Conclusions:**

BMI is a prognostic factor for patients who have gastric cancer with peritoneal dissemination, especially in those who received palliative chemotherapy.

## Background

In China, gastric cancer is the third most common cancer and the second leading cause of cancer-related death [1]. And most patients with gastric cancer are diagnosed with an advanced stage of the disease in China, some even with metastatic disease [2, 3]. Peritoneal dissemination is the most common manifestation for late-stage gastric cancer [4]. The prognosis of patients who have gastric cancer with peritoneal dissemination is very poor, even with the development of targeted therapy and chemotherapy [5, 6]. Palliative gastrectomy is still controversial for this group of patients. In clinic, we believed that the nutritional status is an important factor that impacts the treatment and prognosis of patients who have gastric cancer with peritoneal dissemination.

Body mass index (BMI) is commonly used to assess nutritional status [7]. According to the World Health Organization’s (WHO) guidelines, BMI is divided into three groups with 18.5 and 25 as the cutoff values for a normal BMI level. However, in Asia, a BMI range of 18.5 to 23 is always used to classify people into the underweight group, normal range group, and overweight group [8]. BMI has always been used as an indicator of the status of patients. It had been reported that the BMI would infect the surgical outcomes in colorectal cancer, pancrea cancer, liver cancer, and so on. [9–13]. However, the controversy of the BMI on perioperative morbidity still remained [14–17]. In a report by Pawlik et al. about the BMI and gastric cancer, the overall survival of patients with underweight patients with a BMI <18.5 kg/m^2^ after gastrectomy for cancer was worse than those with BMI higher than 18.5 [18]. Hu et al. reported that a low BMI was associated with more severe postoperative complications and a poor prognosis, compared to patients with a normal BMI [19]. However, there are no reports in the current literature that have examined the relationship between BMI and the prognosis of patients who have gastric cancer with peritoneal dissemination.

The aim of our study was to examine the relationship between BMI and the prognosis of gastric cancer patients who have peritoneal dissemination.

## Methods

### Ethics approval and consent

All patients provided written informed consent for their information to be stored in a hospital database. A separate consent was obtained for the use of this information for research purposes. Study approval was obtained from independent ethics committees at the Sixth Affiliated Hospital of Sun Yat-Sen University. This study was undertaken in accordance with the ethical standards of the World Medical Association Declaration of Helsinki.

### Patients

Between January 2000 and April 2014, a total of 518 patients were histologically proven and diagnosed with gastric adenocarcinoma with peritoneal metastasis in surgery in The Sixth Affiliated Hospital of Sun Yat-sen University and Sun Yat-sen University Cancer Center. Patients were divided into three groups based on their BMI using 18.5 and 23 as the cutoff values. The clinicopathologic characteristics and clinical outcomes of all 518 patients were reviewed.

### Patient inclusion criteria

The inclusion criteria were as follows: (1) WHO performance status of 0 to 1; (2) the patient underwent surgery and had histologically proven gastric adenocarcinoma of the stomach with peritoneal dissemination; (3) no synchronous or metachronous cancers; (4) no history of previous radiotherapy or other treatments, including immunotherapy or traditional Chinese medicine; and (5) no prior gastric surgery.

### Classification of peritoneal seeding

According to the first English edition of the Japanese classification of gastric carcinoma, the degree of peritoneal metastasis was classified as follows: P0, no peritoneal dissemination or seeding; P1, disseminating metastasis to the region directly adjacent to the peritoneum of the stomach (above the transverse colon including the greater omentum); P2, several scattered metastases to the distant peritoneum and ovarian metastasis alone; and P3, numerous metastases to the distant peritoneum [20].

### Follow-up

Following treatment, patients were monitored every month for the first year, every 3 months for the second year, and every 6 months thereafter. Telephone calls and letters were used to follow-up patients who were unable to attend regular follow-up assessments. Complete data were collected for all 523 patients through December 2015.

### Statistical methods

A chi-square test was used to compare the categorical variables between the palliative operative group and the other groups. Student’s *t* test was used to compare continuous variables. Univariate survival analyses were performed using Kaplan-Meier methods. Survival curves were compared using the log-rank test. Analyses were performed using SPSS software v.20.0 (SPSS, Inc., Chicago, IL) for Windows. Statistical significance was defined as *p* < 0.05.

## Results

### Patient demographics

Among the 518 patients with gastric cancer and peritoneal dissemination, 330 underwent non-curative gastrectomy, while 188 patients did not. Their median age was 59 years (range, 18–83). Of these patients, 289 were male and 229 were female. The 1-year overall survival of the entire cohort was 26.0%, with a median survival of 13.2 months. Patients were separated into three groups based on their BMI with the cutoff values of 18.5 and 23. There were 167 patients with a BMI <18.5, 232 with a BMI 18.5–23, and 119 patients with a BMI >23. The patient clinicopathological characteristics are presented in Table 1.

**Table 1:** Clinical pathological data for gastric cancer patients with different BMI groups

Clinical pathological data		Fewer than 18.5 (*n* = 167 cases)		Between 18.5–23 (*n* = 232 cases)		Greater than 23 (*n* = 119 cases)		*p* value
		Cases	%	Cases	%	Cases	%	
Age (years)	Mean	52.6		52.9		53.3		–
	Range	20–85		19–85		29–84		
Sex	Male	95	56.9	122	52.6	72	60.5	
	Female	72	43.1	110	47.4	47	39.5	0.347
Tumor location	Gastric cardia	40	24.0	47	20.3	31	26.1	
	Middle	26	15.6	47	20.3	32	26.9	
	Antrum	63	37.7	92	39.7	36	30.3	
	Total stomach	38	22.8	46	19.8	20	16.8	0.181
Surgery	Gastrectomy	90	53.9	165	71.1	75	63.0	
	No gastrectomy	77	46.1	67	28.9	44	37.0	0.002
Tumor size	<5 cm	50	29.9	91	39.2	47	39.5	
	≥5 and <10 cm	83	49.7	104	44.8	56	47.1	
	≥10 cm	34	20.4	37	15.9	16	13.4	0.249
Serum CEA level	<5 ng/ml	104	67.5	165	73.7	80	70.8	
	≥5 ng/ml	50	32.5	59	26.3	33	29.2	0.433
CA19-9 level	<35 U/ml	91	59.9	151	69.3	72	63.7	
	≥35U/ml	61	40.1	67	30.7	41	36.3	0.166
Seeding grade^a^	P1	28	16.8	66	28.4	42	35.3	
	P2	44	26.3	72	31.0	33	27.7	
	P3	95	56.9	94	40.5	44	37.0	0.001
Multi-site metastasis	Without	97	58.1	181	78.0	81	68.1	
	With	70	41.9	51	22.0	38	31.9	< 0.001
Chemotherapy	Without	77	46.1	68	29.3	36	30.3	
	With	90	53.9	164	70.7	83	69.7	0.001

^a^Peritoneal dissemination grade was divided into P1, P2, and P3 groups under the standard of first English version of Japanese classification of gastric carcinoma

### Univariate analyses of the prognosis of this group of gastric cancer patients with peritoneal dissemination

**Fig. 1:**
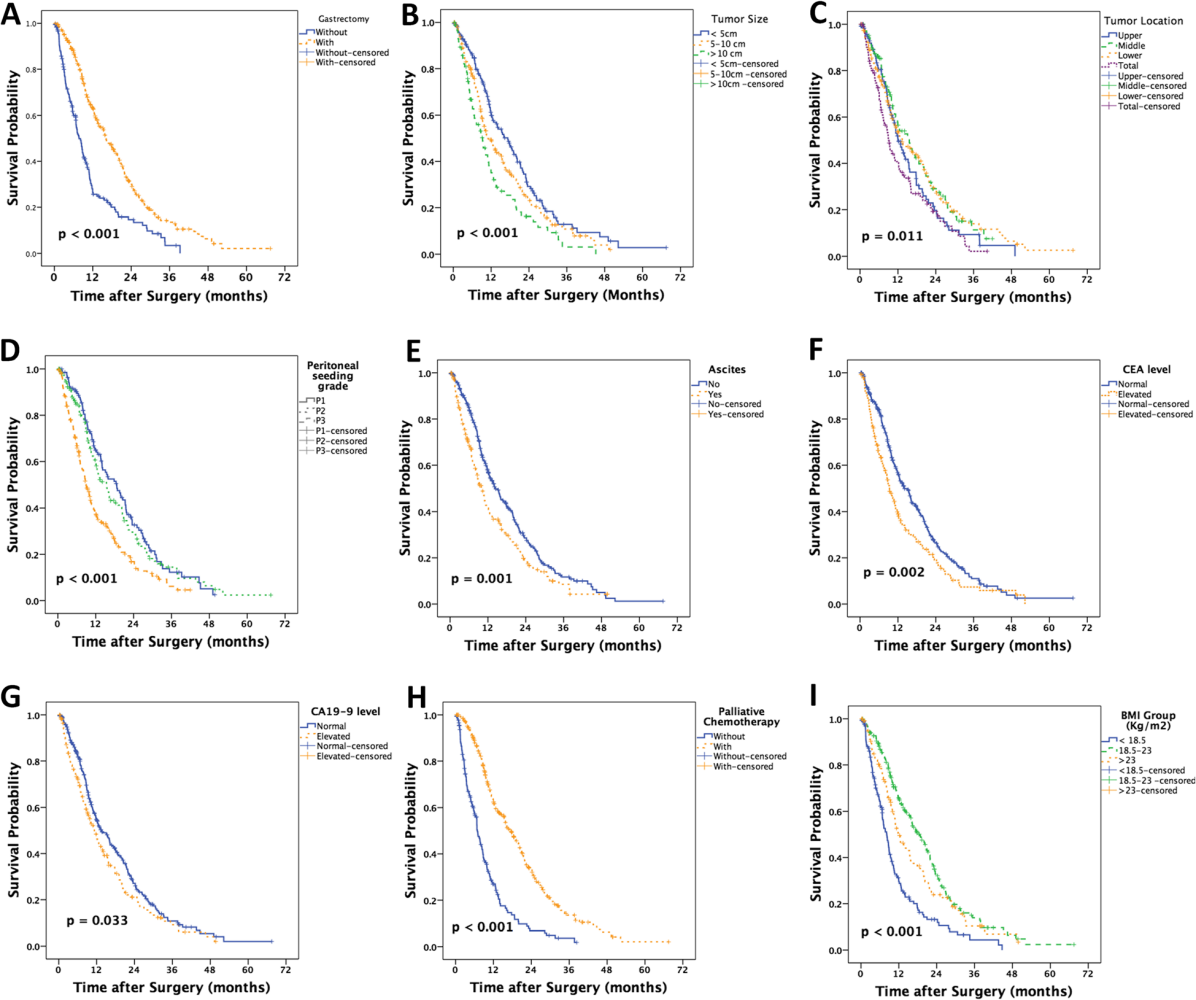
The Kaplan-Meier analysis of the prognosis of this group of gastric cancer patients with peritoneal dissemination. Palliative gastrectomy (**a**), tumor size (**b**), tumor location (**c**), peritoneal seeding grade (**d**), ascites (**e**), serum CEA level (**f**), serum CA19-9 level (**g**), palliative chemotherapy (**h**), and BMI group (**i**) were significant prognostic factors in this cohort

Based on Kaplan-Meier analysis, palliative gastrectomy (*p* < 0.001), tumor size (*p* < 0.001), tumor location (*p* = 0.011), peritoneal seeding grade (*p* < 0.001), ascites (*p* = 0.001), serum CEA level (*p* = 0.002), serum CA19-9 level (*p* = 0.033), palliative chemotherapy (*p* < 0.001), and BMI group (*p* < 0.001) were significant prognostic factors in this cohort, and the results were shown in Fig. 1. As shown in Table 2, the median survival of patients based on BMI were 8.17 months in patients with a BMI less than 18.5, 18.67 months for those with a BMI between 18.5 and 23, and 11.87 months in patients with a BMI greater than 23.

**Table 2:** Univariate analysis of the overall survival in these group gastric cancer patients with peritoneal dissemination

Variables	*n*	1-year survival rate (%)	Median survival (months)	*p* value
All 518 gastric cancer patients with peritoneal dissemination				
Palliative gastrectomy				<0.001
With	330	19	7.33	
Without	188	31	16.13	
Tumor size				<0.001
<5 cm	188	31	17.30	
5 and <10 cm	243	26	11.00	
>10 cm	87	18	9.30	
Tumor location				0.011
Upper	118	23	12.00	
Middle	105	30	15.60	
Lower	191	39	13.33	
Total	104	20	8.97	
Peritoneal seeding grade				<0.001
P1	136	34	18.67	
P2	149	31	15.48	
P3	233	19	8.97	
Ascite				0.001
With	207	20	9.87	
Without	311	31	14.27	
Serum CEA level (ng/ml)				0.002
Normal	349	29	14.03	
Elevated	142	20	9.33	
Serum CA19-9 level (U/ml)				0.033
Normal	314	29	13.53	
Elevated	169	22	11.27	
Palliative chemotherapy				<0.001
Yes	337	35	17.40	
No	181	9	6.90	
BMI group (kg/m^2^)				<0.001
<18.5	167	15	8.17	
18.5–23	232	35	18.67	
>23	119	25	11.87	

### BMI value was an independent prognostic factor of gastric cancer patients with peritoneal dissemination by multivariate analyses

Cox regression model was used to identify the independent prognostic risk factors in patients with gastric cancer and peritoneal seeding. The results revealed that palliative gastrectomy, peritoneal seeding grade, serum CEA level, palliative chemotherapy, and BMI value were independent prognostic risk factors. All of these results are presented in Table 3.

**Table 3:** Multivariate analyses of the overall survival in gastric cancer patients (Cox regression model)

Variable	HR	95% CI	*p* value
OS in gastric cancer patients			
Palliative gastrectomy (Gastrectomy vs. without gastrectomy)^a^	0.689	0.540–0.879	0.003
Tumor size (<5, ≥5, and <10 vs. ≥10 cm)	1.176	0.999–1.383	0.051
Tumor location (Upper, middle, and lower vs. total)	1.080	0.962–1.213	0.192
Ascite (With vs. without)	1.026	0.817–1.288	0.827
Peritoneal seeding grade (P1 and P2 vs. P3)	1.298	1.124–1.500	< 0.001
Serum CEA Level (Normal vs. elevated)	1.424	1.120–1.810	0.004
Serum CA19-9 Level (Normal vs. elevated)	1.133	0.900–1.424	0.287
Palliative chemotherapy (With vs. without)	0.399	0.314–0.506	< 0.001
BMI group (<18.5 and 18.5–23 vs. >23)	0.812	0.686–0.962	0.016

Abbreviations: *OS* overall survival, *HR* hazard ratio, *CI* confidence interval

^a^The factor listed at the last was used as the control level in this Cox regression model

### Univariate analyses and multivariate analysis of the prognosis of gastric cancer patients with peritoneal dissemination without palliative chemotherapy

**Fig. 2:**
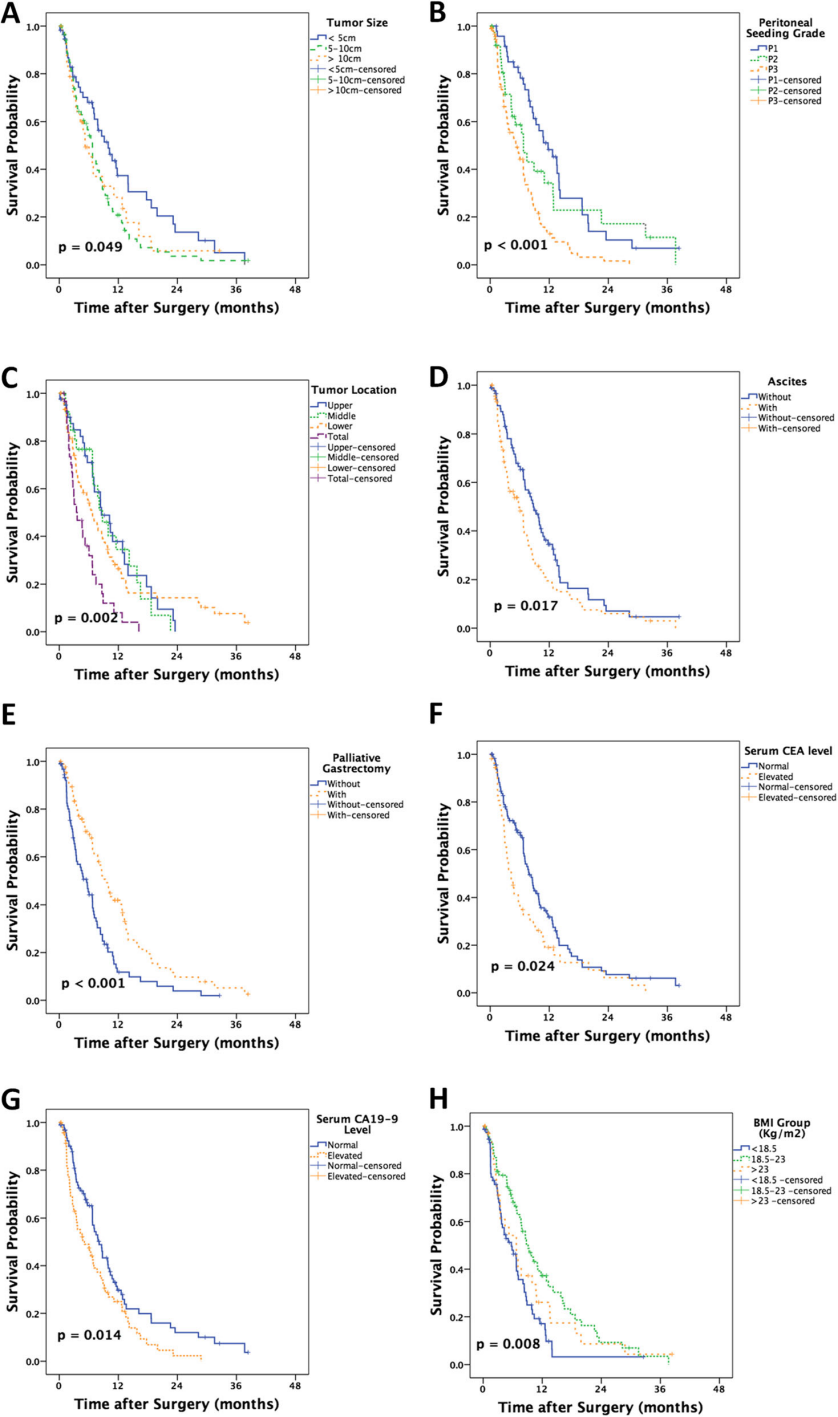
The Kaplan-Meier analysis of the prognosis of this group of gastric cancer patients with peritoneal dissemination without palliative chemotherapy. Palliative gastrectomy (**a**), tumor size (**b**), tumor location (**c**), peritoneal seeding grade (**d**), ascites (**e**), serum CEA level (**f**), serum CA19-9 level (**g**), and BMI group (**h**) were prognostic risk factors

Patients were stratified based on having received palliative chemotherapy or not. Kaplan-Meier analysis showed that palliative gastrectomy (*p* < 0.001), tumor size (*p* = 0.049), tumor location (*p* = 0.002), peritoneal seeding grade (*p* < 0.001), ascites (*p* = 0.017), serum CEA level (*p* = 0.024), serum CA19-9 level (*p* = 0.014), and BMI group (*p* = 0.008) were prognostic risk factors. All of these results are presented in Fig. 2.

Only the tumor location and the peritoneal seeding grade remained independent prognostic factors upon Cox regression analysis, as shown in Table 4.

**Table 4:** Multivariate analyses of the overall survival in these group gastric cancer patients with peritoneal dissemination without palliative chemotherapy (Cox regression model)

Variable	HR	95% CI	*p* value
OS in gastric cancer patients			
Palliative gastrectomy (Gastrectomy vs. without gastrectomy)^a^	0.772	0.516–1.154	0.207
Tumor size (<5, ≥5, and <10 vs. ≥10 cm)	1.016	0.771–1.339	0.910
Tumor location (Upper, middle, and lower vs. total)	1.314	1.070–1.614	0.009
Ascite (With vs. without)	0.969	0.653–1.438	0.877
Peritoneal seeding grade (P1 and P2 vs. P3)	1.507	1.178–1.928	0.001
Serum CEA Level (Normal vs. elevated)	1.356	0.915–2.011	0.129
Serum CA19-9 Level (Normal vs. elevated)	1.384	0.943–2.031	0.097
BMI group (<18.5 and 18.5–23 vs. >23)	0.871	0.654–1.161	0.346

Abbreviations: *OS* overall survival, *HR* hazard ratio, *CI* confidence interval

^a^The factor listed at the last was used as the control level in this Cox regression model

### Univariate analyses and multivariate analysis of the prognoses of gastric cancer patients with peritoneal dissemination with palliative chemotherapy

**Fig. 3:**
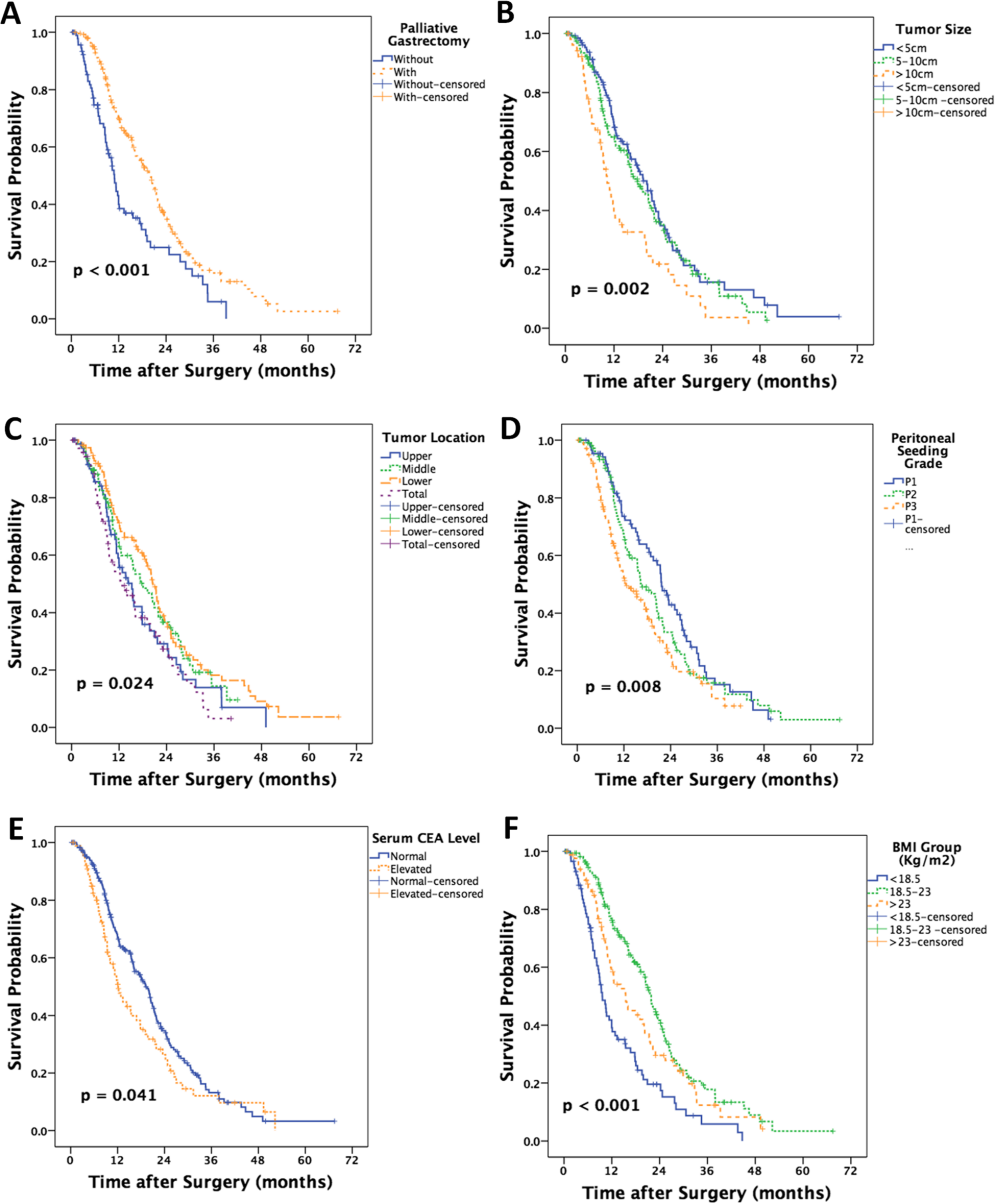
The Kaplan-Meier analysis of the prognosis of this group of gastric cancer patients with peritoneal dissemination underwent palliative chemotherapy. Palliative gastrectomy (**a**), tumor size (**b**), tumor location (**c**), peritoneal seeding grade (**d**), serum CEA level (**e**), and BMI group (**f**) were risk factors

As shown in Fig. 3, 337 patients received palliative chemotherapy in this cohort. Results from Kaplan-Meier analysis showed that the palliative gastrectomy (*p* < 0.001), tumor size (*p* = 0.002), tumor location (*p* = 0.024), peritoneal seeding grade (*p* = 0.008), serum CEA level (*p* = 0.041), and BMI group (*p* < 0.001) were risk factors.

Multivariate analysis showed that palliative gastrectomy, serum CEA level, and BMI group were independent prognostic factors, as showed in Table 5.

**Table 5:** Multivariate analyses of the overall survival in these group gastric cancer patients with peritoneal dissemination with palliative chemotherapy (Cox regression model)

Variable	HR	95% CI	*p* value
OS in gastric cancer patients			
Palliative gastrectomy (Gastrectomy vs. without gastrectomy)^a^	0.652	0.477–0.893	0.008
Tumor size (<5, ≥5, and <10 vs. ≥10 cm)	1.265	1.027–1.559	0.027
Tumor location (Upper, middle, and lower vs. total)	0.972	0.844–1.118	0.689
Peritoneal seeding grade (P1 and P2 vs. P3)	1.172	0.982–1.398	0.079
Serum CEA level (Normal vs. elevated)	1.413	1.045–1.912	0.025
BMI group (<18.5 and 18.5–23 vs. >23)	0.783	0.636–0.964	0.021

Abbreviations: *OS* overall survival, *HR* hazard ratio, *CI* confidence interval

^a^The factor listed at the last was used as the control level in this Cox regression model

## Discussion

With the increasing prevalence of obesity that occurred in China and worldwide [21], few recent reports have concluded that some classes of obesity can be considered “healthy.” Findings from our study show that BMI value was an independent prognostic factor for patients who have gastric carcinoma with peritoneal seeding. Patients with low BMI as well as a high BMI had a worse survival rate compared with patients who had a BMI within normal range. Previous studies have reported that patients with higher BMI would be at higher risk for perioperative morbidity after major abdominal cancer surgery [16, 22]. In the study from Memorial Sloan-Kettering Cancer Center, the results showed that higher BMI of the patients (higher than 25) would bring longer operative time, fewer lymph nodes, and higher complications [15]. In our study, a higher ratio of the patients with a normal BMI underwent palliative gastrectomy, which is an important independent prognostic factor, in addition to palliative chemotherapy.

However, the function of palliative gastrectomy remains controversial, especially for the patients with peritoneal dissemination. Some studies have suggested that palliative gastrectomy may improve survival without increasing morbidity and mortality [23, 24], while other reports have contradicted this suggestion [2, 25]. Selection of patients for palliative gastrectomy remains controversial for both surgeons and oncologists. In our studies, we stratified the 518 patients based on receipt of palliative chemotherapy to eliminate the influences of the palliative chemotherapy. Among the patients who did not receive palliative chemotherapy, neither palliative gastrectomy nor BMI was independent prognostic factors. Conversely, among patients who received palliative chemotherapy, both palliative gastrectomy and BMI were independent prognostic factors. This finding implies that patients with a normal BMI who receive palliative chemotherapy may benefit from gastrectomy.

As a retrospective study, there were confounding factors that may have influenced the statistical analyses and conclusions. BMI is an important factor that reflects the nutritional status and is correlated with postoperative complications and long-term survival of gastric cancer. Findings from this study suggest that gastric cancer patients with a normal BMI may benefit from palliative gastrectomy combined with chemotherapy. Additional experiments and clinical trials are needed to validate the important value of BMI on the prognosis of gastric cancer patients with peritoneal dissemination.

## Conclusions

BMI is a prognostic factor for patients who have gastric cancer with peritoneal dissemination, especially in those who received palliative chemotherapy. BMI can be used to predict the effect of palliative chemotherapy on these patients.

## Abbreviations

BMI: Body mass index (BMI)
OS: Overall survival
WHO: World Health Organization
